# Survival Outcomes in Squamous Cell Carcinoma of the External Auditory Canal: A Systematic Review and Meta-Analysis

**DOI:** 10.3390/jcm12072490

**Published:** 2023-03-24

**Authors:** Diego Cazzador, Leonardo Franz, Giulia Tealdo, Andrea Luigi Camillo Carobbio, Maria Ferraro, Antonio Mazzoni, Gino Marioni, Elisabetta Zanoletti

**Affiliations:** 1 Otolaryngology Section, Department of Neuroscience DNS, University of Padova, 35100 Padova, Italy; 2 Phoniatrics and Audiology Unit, Department of Neuroscience DNS, University of Padova, 31100 Treviso, Italy

**Keywords:** temporal bone carcinoma, external auditory canal, squamous cell carcinoma, survival, meta-analysis, systematic review

## Abstract

Squamous cell carcinomas (SCC) of the external auditory canal (EAC) are rare tumors representing a surgical challenge. Current knowledge is based largely on case series; thus, the level of evidence is weak. This study sought to systematically review the available SCC of the EAC literature and to identify risk factors for overall survival (OS) and disease-specific survival (DSS). A systematic review and meta-analysis of papers searched up to December 2022 through PubMed, Scopus, Web of Science, and Cochrane Library databases was conducted. Quality assessment of the eligible studies was done according to the Newcastle-Ottawa Scale. Pooled univariate and multivariable analyses and meta-analysis using a random-effects or fixed-effects Mantel-Haenszel model were performed. Fifteen articles (282 patients) met the inclusion criteria and were included in the quantitative analysis. The pooled multivariable analysis revealed cT3 and cT4 as independent prognostic factors for OS (*p* = 0.005, and *p* < 0.001, respectively) and DSS (*p* = 0.002, and *p* < 0.001, respectively). Local recurrence rate was 32.3%. The meta-analysis estimated significantly higher odds ratios for advanced T categories, than cT1-T2 tumors for OS and DSS (OR = 3.55; 95% CI, 1.93–6.52, and OR = 3.73; 95% CI, 2.00–6.97, respectively). In conclusion, locally advanced tumors were associated with poor prognosis. Poor outcomes mostly occurred due to local recurrence.

## 1. Introduction

Malignancies of the temporal bone are rare tumors with a locally aggressive behavior. They represent about 0.2% of all head and neck malignancies, with an incidence of 1–6 cases per million population [1,2]. Squamous cell carcinoma (SSC) is the most common malignant histotype in the temporal bone. Advanced SCC of the temporal bone (TBSCC) originating from the external auditory canal (EAC) is characterized by a dismal prognosis, mainly due to the local invasive behavior, the undetected ways of microscopic spreading, and the complexity of the anatomy of this region [3,4], which often leads to inadequate resections. Commonly reported negative prognostic factors include advanced tumor stage at presentation, nodal involvement, facial paralysis at diagnosis, non-anterior spread of the tumor (i.e., dura, petrous bone vs. anterior soft tissues invasion), and positive margins [3]. The level of evidence in the literature is weak since the incidence of this disease is low, and most of the available results were based on case series of limited sample size. In addition, several investigations included different malignant histotypes in the same series [5,6,7,8,9], adding a confounding factor in outcome assessment and reporting.

Several classifications have been proposed for staging TBSCC [10,11,12,13,14], and the lack of a universally accepted system remains a crucial issue, thus making it difficult to compare outcomes and assess the efficacy of treatments. TBSCC management has been a debated topic in the last years, and no definitive consensus has been reached to date on the therapeutic approach to SCC of the EAC. As recently outlined [4], persistent pitfalls are still evident in diagnosis, tumor assessment at pathology, treatment (en bloc vs. piece-meal resection), appropriateness of elective neck dissection and parotidectomy, and role of adjuvant therapy [15,16,17,18].

The main aim of this study was to systematically review the available literature reporting survival outcomes in SCC of the EAC. A meta-analysis was conducted to identify risk factors for overall survival (OS) and disease-specific survival (DSS). A pooled data analysis was also performed to assess time-related independent prognostic factors of survival.

## 2. Materials and Methods

### 2.1. Search Strategy

A systematic literature review and meta-analysis were performed in accordance with the Preferred Reporting Items for Systematic Reviews and Meta-Analysis (PRISMA) statement [19] (PRISMA Checklist available in Table S1, Supplementary Materials).

The electronic databases PubMed, Scopus, Web of Science, and the Cochrane Library were searched from database inception to 2 December 2022 to identify studies that reported the survival outcomes in SCC of the EAC. A search strategy without any filters was used containing the terms “external auditory canal”, “temporal bone”, “squamous cell carcinoma”, “treatment”, “surgery”, and “neck dissection”. The reference lists of the retrieved articles were screened for additional relevant publications. After duplicates removal, four reviewers (D.C., G.T., A.L.C.C., M.F.) independently screened all titles and abstracts and then evaluated the full texts of the eligible articles based on the inclusion criteria. Any disagreement between the reviewers was resolved through discussion with all authors to reach a consensus.

### 2.2. Selection Criteria

Studies were deemed eligible when they met the following inclusion criteria: (i) confirmed pathological diagnosis of SCC of the EAC; (ii) tumors staged according to the modified Pittsburgh classification [11]; (iii) tumors primarily treated with surgery; and (iv) availability of complete or extractable data on tumor features, outcomes, and survival. The exclusion criteria were as follows: (i) retrospective series with less than five patients; (ii) tumor origin in the middle ear or subsites of the temporal bone other than EAC; (iii) patients treated primarily with radiotherapy or chemoradiation; (iv) lack of relevant data; (v) non-original studies (i.e., reviews, recommendations, letters, editorials, and book chapters); and (vi) non-English studies. Studies presenting aggregated data on tumor-related characteristics and survival outcomes were excluded. The papers were rigorously screened for duplicate data. To rule out confounding variables, the selected articles included only primary SCC tumors of the EAC surgically treated with curative intent, homogeneously staged according to the modified Pittsburgh staging system.

### 2.3. Data Extraction and Quality Assessment

Extracted data were collected in an electronic database including first author, year of publication, country of origin, study design, total sample size, number of patients included in the meta-analysis according to the inclusion criteria, age at diagnosis, sex, cT category, nodal status, type of resection, margin status, parotidectomy, neck dissection, adjuvant treatments, pattern of recurrence, survival outcomes, and follow-up time. The staging system applied was homogeneous for all the patients, namely the modified Pittsburgh classification.

The quality of the studies eligible for inclusion was assessed according to the Newcastle-Ottawa scale (NOS) for cohort studies [20]. Two items in the scale (Selection of the non-exposed cohort and Comparability) were not considered since none of the included studies presented a control group, thus the maximum NOS score was considered 6 stars instead of 9. Studies with NOS scores 0–2, 3–4 and 5–6 were considered as low, moderate, and high quality, respectively. Two reviewers (A.L.C.C. and M.F.) independently evaluated the papers and any disagreement was resolved by consensus.

### 2.4. Statistical Analysis

Descriptive statistics were calculated for the pooled cohort as means and standard deviations (SD) for continuous variables and as frequencies and percentages for categorical ones. OS and DSS for the pooled sample were determined calculating the Kaplan–Meier estimator of survival. Log-rank test was used to compare the outcomes between different cT categories and N status. Univariate analysis of hazard ratios (HRs) was calculated for the variables cT category, N status, type of temporal bone resection, pathological margin status, and adjuvant treatment for OS and DSS. Multivariable analysis with Cox proportional hazards model included cT category and N status for both independent variables of survival. Type of temporal bone resection and adjuvant treatment were excluded due to multicollinearity after variance inflation factor (VIF) calculation; pathological margin status was excluded due to high numbers of missing data.

The meta-analysis was performed with Stata Meta Suite, Stata 16.1 (StataCorp LLC, College Station, TX, USA), using a random-effects or a fixed-effects Mantel-Haenszel model, as appropriate. The overall effect sizes for binary outcomes were reported as odd ratios (OR) and 95% confidence intervals (95% CI) and were estimated and outlined as forest plots. Heterogeneity was assessed through the I^2^ statistics. The amount of heterogeneity was interpreted according to Higgins [21] as follows: I^2^ values between 25% and 50% (low heterogeneity), I^2^ between 50% and 75% (moderate heterogeneity), and I^2^ > 75% (high heterogeneity). Publication bias was evaluated using funnel plots, and small-study effect was assessed with the regression-based Egger test. Statistical significance was set at *p* < 0.05. Statistical analyses were performed with Stata 16.1 (StataCorp LLC, College Station, TX, USA).

## 3. Results

### 3.1. Search Results and Quality Assessment

**Figure 1:**
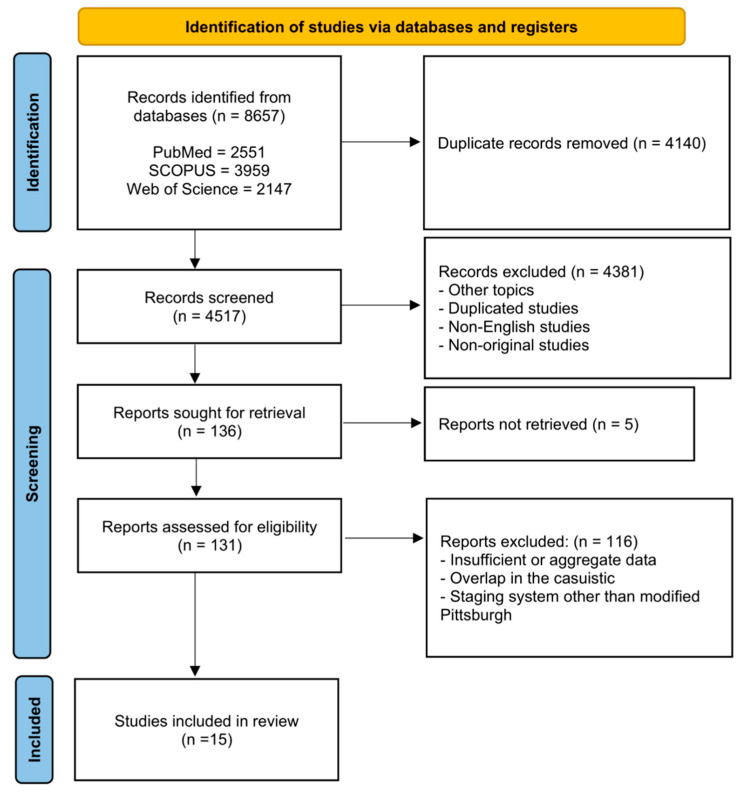
PRISMA flowchart of the study inclusion process. n = number.

The bibliographic research yielded 8657 articles. After duplicates removal and excluding 4381 records due to the aforementioned criteria, 131 full texts were examined for eligibility (Figure 1). Of these, 116 were excluded as they presented insufficient or aggregate data, staging systems other than the modified Pittsburgh’s, or overlapping data with series of patients already included. Finally, 15 articles fulfilled the inclusion criteria [4,22,23,24,25,26,27,28,29,30,31,32,33,34,35].

Seven studies (47%) reported a NOS score of 6, and five studies (33%) had 5. Scores < 5 were observed in three studies (20%); of these, two and one papers were scored 4 and 3, respectively. Comprehensively, the median overall score was 5. A detailed description of the quality of included studies is reported in Table S2 (Supplementary Materials). A high-quality rating was reached by 12 studies (80%). None of the included studies was classified as low quality.

### 3.2. Characteristics of the Studies and Pooled Analysis

All the 15 studies included in the meta-analysis [4,22,23,24,25,26,27,28,29,30,31,32,33,34,35] were retrospective case-series published between 2003 and 2022 (Table 1). The median number of patients per study was 21 (range 10–52). The eligible studies enrolled 282 patients with a mean age of 61.2 ± 13.2 years. Gender was reported in 13 studies, including 131 males (54.8%) and 108 females (45.2%). Pooled data on demographics, tumor characteristics and survival outcomes are reported in Table 2.

At diagnosis, most patients presented advanced tumors (cT3-cT4, 57.1%) without nodal metastases (N0, 85.1%). Lateral temporal bone resection (LTBR) was performed in 54.6% of cases, concomitant parotidectomy in 52.8%, and neck dissection in 35.1%. Five studies (33.3%) did not report data on the status of surgical margins. At a mean follow-up time of 50.8 ± 51.8 months, 28.0% of patients were dead from the disease and 5.3% alive with the disease.

**Figure 2:**
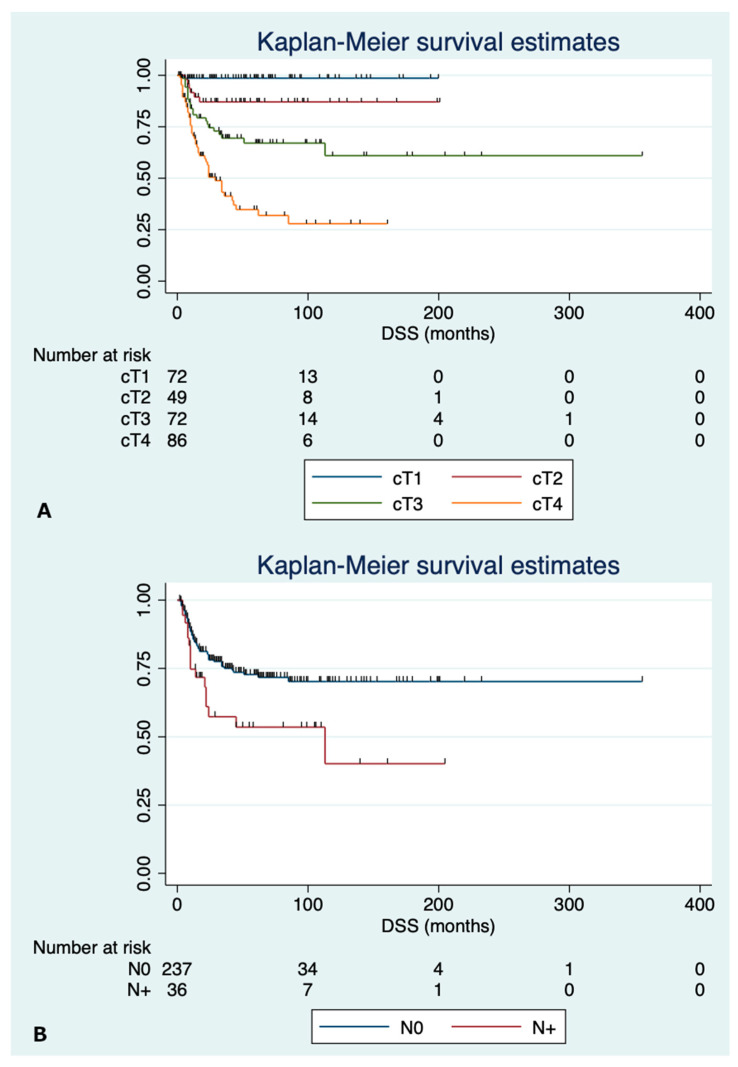
Disease-specific survival according to cT category (**A**) and N status (**B**).

Pooled 3-, 5-year OS calculated for 279 patients was 65.7% and 61.8%, respectively, with median OS time of 147.0 months. DSS was 72.0% and 69.0% at 3- and 5-year follow-up, respectively. Kaplan–Meier curves are plotted in Figure S1 (Supplementary Material). 5-year OS significantly decreased according to cT category (*p* < 0.001), being 82.6%, 80.2%, 62.2%, and 32.0% for cT1, cT2, cT3, and cT4 tumor categories, respectively (Figure S2a, Supplementary Material). Analogous behavior was found for DSS, where cT1, cT2, cT3, and cT4 tumor categories presented a survival rate of 98.6%, 87.1%, 67.1%, and 34.8%, respectively (Figure 2A). As for N status, both OS and DSS were significantly lower in N+ patients, with 64.9% and 48.8% 5-year OS in N0 and N+ patients, respectively (*p* = 0.029), (Figure S2b, Supplementary Material), and 72.7% and 53.6% 5-year DSS (*p* = 0.014) (Figure 2B).

Results of pooled univariable and multivariable analyses are reported in Table 3 and Table 4. Advanced tumor categories (cT3 and cT4) were independent prognostic factors for OS (HR = 2.65 95% CI, 1.34–5.22; HR = 5.51 95% CI, 2.76–10.5, respectively) and DSS (HR = 23.8 95% CI, 3.20–177.8; HR = 56.0 95% CI, 7.67–409.1, respectively).

### 3.3. Meta-Analysis for cT Category

**Figure 3:**
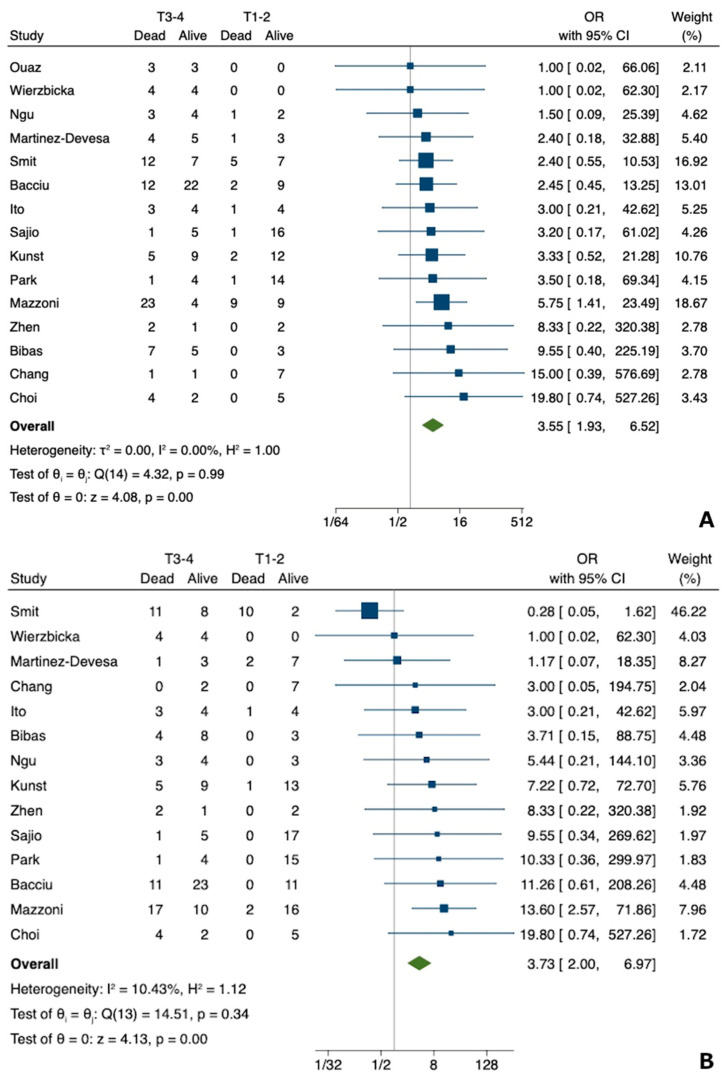
Forest plot for the odds ratio of cT category and survival ((**A**) overall survival [4,22,23,24,25,26,27,28,29,30,31,32,33,34,35]; and (**B**) disease specific survival [4,22,23,24,25,26,27,28,30,31,32,33,34,35]) in temporal bone squamous cell carcinoma of the external auditory canal.

Based on the 15 studies included [4,22,23,24,25,26,27,28,29,30,31,32,33,34,35], the odds ratio of death from any cause for T3-4 group, as compared with T1-2 patients, was 3.55 (95% CI, 1.93–6.52), based on a random-effects model (see also Figure 3a). No significant heterogeneity was found (I^2^ = 0.00%, *p* = 0.99). The funnel plot was symmetrical (Figure S3a, Supplementary Materials), ruling out any suspected publication bias. No significant small-study effect was detected either (regression-based Egger test: *p* = 0.856). Regarding death from disease, in 14 studies [4,22,23,24,25,26,27,28,30,31,32,33,34,35] enrolling 276 patients, the odds ratio for T3-4 group, as compared with T1-2 patients, was 3.73 (95% CI, 2.00–6.97), based on a fixed-effects Mantel-Haenszel model (see also Figure 3b). A small, non-significant heterogeneity was found (I^2^ = 10.43%, *p* = 0.34). In this case, the funnel plot was symmetrical (Figure S3b, Supplementary Materials), and no significant small-study effect was noticeable (regression-based Egger test: *p* = 0.443).

### 3.4. Meta-Analysis for N Status

**Figure 4:**
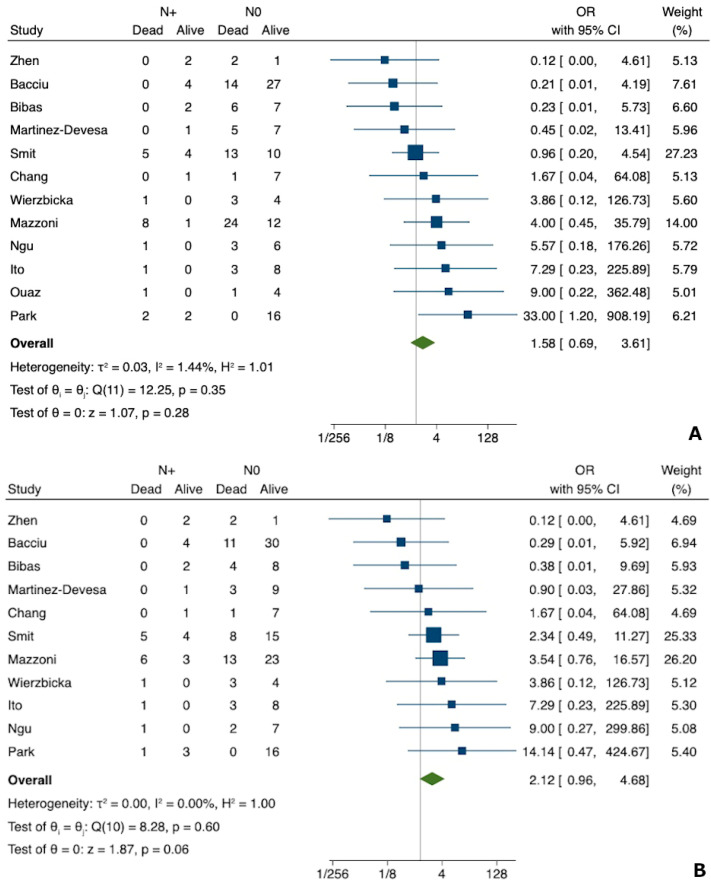
Forest plot for the odds ratio of N status and survival ((**A**)—overall survival [4,23,24,26,27,28,29,30,31,32,33,35]; (**B**)—disease specific survival [4,23,24,26,27,28,30,31,32,33,35]) in temporal bone squamous cell carcinoma of the external auditory canal.

Based on 12 studies that reported N status in 220 individuals [4,23,24,26,27,28,29,30,31,32,33,35], the odds ratio of death from any cause for N+ group, as compared with N0 cases, was 1.58 (95% CI, 0.69–3.61), based on a random-effects model (see also Figure 4a). No significant heterogeneity was found (I^2^ = 1.44%, *p* = 0.35). The funnel plot was symmetrical (Figure S3c, Supplementary Materials), ruling out any suspected publication bias. No significant small-study effect was detected either (regression-based Egger test: *p* = 0.712).

N status and death from disease outcomes were reported in 11 studies (214 patients) [4,23,24,26,27,28,30,31,32,33,35]; the odds ratio for the N+ group, as compared with N0 patients, was 2.12 (95% CI, 0.96–4.68), based on a random-effects model (see also Figure 4b). No significant heterogeneity was found (I^2^ = 0.00%, *p* = 0.60). Furthermore, in this case, the funnel plot was symmetrical (Figure S3d, Supplementary Materials), and no significant small-study effect was noticeable (regression-based Egger test: *p* = 0.508).

## 4. Discussion

### 4.1. Summary of Findings

Our meta-analysis of 15 studies including patients primarily treated with surgery for SCC of the EAC estimated significantly higher odds ratios for advanced T categories than cT1-T2 tumors for OS and DSS (OR = 3.55; 95% CI, 1.93–6.52 and OR = 3.73; 95% CI, 2.00–6.97, respectively). Conversely, N status did not significantly influence survival (OR = 1.58; 95% CI, 0.69–3.61 for OS, and OR = 2.12; 95% CI, 0.96–4.68 for DSS).

### 4.2. Comparison with Other Studies

To our knowledge, this is the first systematic review with meta-analysis on recurrence and survival outcomes of TBSCC that included only primary tumors of the EAC exclusively classified with the modified Pittsburgh staging system.

Recently, McCracken et al. [36] published a systematic review on patients with TBSCC who underwent temporal bone resection (TBR) with curative intent.

In contrast to our review, they included both primary TBSCC (originating from multiple subsites) and metastatic SCC to the temporal bone, which were classified with the Pittsburgh or AJCC staging system. In agreement with our results, they obtained a worse survival in patients with advanced-stage disease. In addition, the authors focused on the relation between type of treatment and survival: patients undergoing subtotal TBR or total TBR showed poorer survival and recurrence outcomes compared to patients receiving LTBR, with a 97% increased risk of mortality. This is understandable since STBR and TTBR are usually performed in advanced cases.

Most of the available reviews on TBSCC outcomes are narrative. In 2019, Lovin and Gidley’s [1] TBSCC literature review found that the 5-year DSS rates for T1-2 and T3-4 tumors ranged from 92% to 100% and from 48% to 65%, respectively. Lechner et al. [37] identified 16 case series of TBSCCs including 708 individuals, of whom 578 had undergone surgery. The authors reported that survival correlated with disease stage in all studies. Other identified prognostic variables included tumor grade, nodal involvement, positive surgical margins, dural invasion, and facial nerve involvement.

As for the management of the neck in SCC of the EAC, elective neck dissection for cN0 patients is still under debate. A recent systematic review and meta-analysis by Borsetto et al. [38] estimated that the rate of occult lymph node metastases in cN0 TBSCC was 14%, with specific subgroup rates of 21% for T3 tumors and 18% for T4 tumors. Metastases were predominantly localized at level II. For these reasons, the authors suggested performing a selective neck dissection of neck levels II and III in locally advanced, cN0 TBSCC. In their retrospective analysis on 63 patients with SCC of the EAC, Kiyokawa et al. [18] encountered 18 cN+ cases. The distribution of node metastases mostly involved level II (56%), the parotid gland (39%), and nodes of the preauricular area (28%). The authors discouraged the need for elective neck dissection in locally advanced cN0 tumors without free flap reconstruction, since in their experience nodal recurrences were successfully controlled through neck dissection when needed. Likewise, elective neck dissection of levels Ib to III was suggested for cT3/T4cN0 patients with planned free flap reconstruction.

### 4.3. Principles of Treatment in SCC of the EAC

SCC of the EAC represents a rare and challenging condition to be managed. Its locally aggressive behavior (32.3% of local recurrence recorded in the pooled analysis of the 282 cases included) dictates a radical surgical excision with free surgical margins on pathological examination. Focusing on the surgical technique, two options are encountered in the literature: piecemeal and *en bloc* resections. The latter is technically demanding and requires highly experienced surgeons. This type of resection removes the tumor following an anatomical plane of dissection through normal tissue, thus allowing pathological margin determination, and tumor extent evaluation for pT staging. The en bloc principle of resection, as standardized by Mazzoni et al. [15,39], can be applied to any tumor category in LTBR and STBR. En bloc margin-negative resection was proved to be a reliable treatment strategy in TBSCC even for locally advanced tumors [34,40].

Superficial parotidectomy was advocated to be associated with surgery for early-stage tumors and total parotidectomy for advanced-stage disease [15]. Although the data is too limited to draw any conclusions on the role of elective neck dissection, increasing evidence indicates that levels Ib to III in cN0 cases should be included [18,38,41].

Adjuvant RT is generally indicated for advanced TBSCC (T3–T4), or in the case of aggressive pathological features, such as perineural invasion, close (<5 mm) or positive margins, lymph node metastases, or extracapsular spread [16,42]. Some authors report usually treating the tumor bed with an RT boost up to a total dose of 66–70 Gy to gain local control in cases of positive surgical margins or residual tumor after surgery [43,44]. It is well known that the prognosis for patients with TBSCC treated with chemo-radiotherapy alone is unsatisfactory [2]. Neoadjuvant chemotherapy has recently been described as an option to downstage borderline resectable tumors to aid margin free excision [45]. The role of chemotherapy has not been fully defined, but its application, as well as other non-surgical palliative treatments (radiotherapy, or specialist palliative care) are recommended for patients with loco-regionally advanced recurrent TBSCC [46]. A combination of cisplatin with 5-fluorouracil (5FU) has been suggested as the most effective association for palliation: it was not associated with any significant improvement in the survival rate but did improve pain control [16,25].

### 4.4. Clinical Relevance

Our results confirmed the evidence that advanced TBSCC presents a worse prognosis than early tumors (cT1-2). Even if several potential prognostic factors have been reported in the literature [37], such as tumor grade, nodal involvement, positive surgical margins, dural invasion, and facial nerve involvement, in our study, the cT stage appears to be the only independent prognostic factor impacting prognosis. Such findings underline that early diagnosis is crucial to improve TBSCC survival.

Unfortunately, TBSCC of the EAC are often diagnosed late; in the literature, average time from symptom presentation to diagnosis ranges from 12.4 months to 3.9 years [47]. Consequently, the rate of advanced tumors at diagnosis is high; in our pooled analysis, we found 57.1% of cT3-4 tumors at diagnosis. One of the main reasons for the diagnostic delay in TBSCC is probably the non-specificity of the presenting symptoms. Cancers of the EAC often mimic a chronic infective condition. In 2013, Zhang et al. [48] conducted a retrospective study of misdiagnosed EAC tumors. Among 18 cases, 6 were initially clinically diagnosed as otitis media, 5 as otitis externa, 2 as external auditory canal cholesteatoma, and other patients received a first diagnosis of EAC stenosis, ear neuralgia, EAC furuncle, EAC benign tumor, and pre-auricular fistula. According to this evidence, a biopsy should always be considered when otorrhea and/or otorrhagia are resistant to treatments. Moreover, when clinical suspicion of TBSCC of the EAC is high, micro-otoscopy with extensive, multiple biopsies is essential to increase the probability of achieving the correct diagnosis of TBSCC. In fact, neoplastic tissue often coexists with granulation tissue [2,16,48]. Additionally, preoperative imaging acquisition combining high-resolution temporal bone computed tomography with contrast-enhanced magnetic resonance imaging is crucial for the assessment of tumor size and extent. The former is the most accurate radiological method for examining cancerous bone erosion, whereas MRI defines the boundaries between the tumor and surrounding tissues [46,49]. The role of CT and MRI is also important not only in preoperative staging and treatment planning, but also in follow-up of SCCs of the EAC. Given the high rate of local recurrences in the early postoperative period, several authors recommended a strict follow-up schedule including at least clinical examination and contrast-enhanced head and neck MRI every two months in the 1st year and every four months in the 2nd year; clinical examination and MRI was recommended every six months in the 3rd to fifth years [46,49].

The highest accuracy in the histopathological definition of the tumor features is also advocated in order to properly stage EAC SCC and indicate, if necessary, adjuvant treatments. En bloc surgery enables serial histopathological studies of the surgical specimen, which allows correct pT classification and a meaningful correlation between pT, surgical procedure, and outcomes [4,15]. This is probably the way to improve and redefine our understanding of EAC SCC.

### 4.5. Strengths and Limitations of the Study

The limitations of this study go beyond the low number of cases reporting adequate survival data in TBSCC. They reflect the persistent pitfalls of the current debate on TBSCC prognostic factors, where the original bias of defective data provided by imaging and histopathology promoted defective classifications and inadequate assessment of outcomes. This basic lack of knowledge is the preliminary condition that must be borne in mind. Even among the cases of the selected series, there was a lack of information on possible prognosticators of survival as margin status, facial nerve involvement, and direction of tumor spread. In addition, other sources of limitation of our meta-analysis were: (i) non-homogeneous surgical management of the tumor, considering surgical techniques of en bloc vs. piecemeal resections; (ii) non-homogeneous treatment of the neck, and data reporting regarding N status; (iii) not standardized adjuvant treatment among the studies included; and (iv) lack of review registration and protocol. From a statistical perspective, another limitation was due to the inclusion of only retrospective case-series in the analyses. Direct evidence, especially in orphan diseases such as TBSCC of the EAC, are hardly achievable through retrospective research studies. As such, given the rarity of the disease, prospective studies are unavailable in the literature to date. Efforts to create sufficient evidence and evidence-based practice and standards in rare diseases include international collaborations and high-level evidence studies such as systematic reviews with meta-analysis, meta-regressions, and network meta-analysis [50,51,52]. Unfortunately, non-homogeneous data in the single series retrieved regarding adjuvant treatment or neck dissection precluded the possibility of conducting a subgroup analysis or a meta-regression.

This analysis’ strengths included the systematic and quantitative assessment of the role of cT category and N status in SCC of the EAC, completed by a pooled multivariable analysis of risk factors. It should be noted that our strict inclusion and exclusion criteria ensured a homogeneous pooled patients’ sample according to histology, site of tumor origin, and tumor staging. Given the high rates of inconsistencies between the staging systems adopted worldwide, which may affect prognostic stratification [53], only articles reporting the modified Pittsburgh staging system were deemed eligible for inclusion.

## 5. Conclusions

Locally advanced SCC of the EAC (cT3 and cT4) were independent predictors of OS and DSS and were associated with poor prognosis on meta-analysis. Poor outcomes mostly occurred due to the high rate of local recurrence. Nodal involvement of the neck did not independently predict survival. These results prompt the need for a primary radical tumor excision to reduce the risk of local recurrence. Further analyses of prognostic factors based on larger, prospective, multicentric cohorts are advocated.

## Figures and Tables

**Table 1:** Characteristics of the articles included in the meta-analysis.

Author	Year of Publication	Country	Total Number Patients	Number of Patients Included in the Meta-Analysis *	Study Design
Choi et al. [22]	2003	Korea	21	11	CS
Martinez-Devesa et al. [23]	2007	UK	27	13	CS
Bibas et al. [24]	2008	UK	17	15	CS
Kunst et al. [25]	2008	Netherlands–France	28	28	CS
Chang et al. [26]	2009	China	12	9	CS
Ito et al. [27]	2009	Japan	16	12	CS
Bacciu et al. [28]	2013	Italy	45	45	CS
Ouaz et al. [29]	2013	France	10	6	CS
Zhen et al. [30]	2014	China	16	5	CS
Wierzbicka et al. [31]	2016	Poland	20	8	CS
Park et al. [32]	2018	Korea	31	20	CS
Ngu et al. [33]	2021	Malaysia	10	10	CS
Sajio et al. [34]	2021	Japan	52	23	CS
Smit et al. [35]	2021	Netherlands	49	32	CS
Mazzoni et al. [4]	2022	Italy	45	45	CS

* According to the inclusion/exclusion criteria. CS = Case series.

**Table 2:** Pooled data of patients with external auditory canal squamous cell carcinoma.

Variable	N (%)
Patients	282
Age, mean ± SD, years	61.2 ± 13.2
Sex Male Female Missing	131 (46.5) 108 (38.3) 43 (15.2)
cT category 1 2 3 4	72 (25.5) 49 (17.4) 75 (26.6) 86 (30.5)
N category N0 N+ Missing	240 (85.1) 36 (12.8) 6 (2.1)
Type of surgical resection SR LTBR STBR TTBR	31 (11.0) 154 (54.6) 87 (30.9) 10 (3.5)
Neck dissection No Yes Missing	149 (52.8) 99 (35.1) 34 (12.1)
Parotidectomy No Yes Missing	117 (41.5) 149 (52.8) 16 (5.7)
Surgical margins Negative Positive Missing	86 (30.5) 72 (25.5) 124 (44.0)
Adjuvant treatments None RT CRT Missing	80 (28.4) 195 (69.1) 6 (2.1) 1 (0.4)
Patterns of recurrence Local Nodal Distant	91 (32.3) 11 (3.9) 2 (0.7)
Survival NED AWD DOD DOC	159 (56.4) 15 (5.3) 79 (28.0) 29 (10.3)
Survival time, mean ± SD, months	50.8 ± 51.8

SD = standard deviation; SR = sleeve resection; LTBR = lateral temporal bone resection; STBR = subtotal temporal bone resection; TTBR = total temporal bone resection; RT = radiotherapy; CRT = chemoradiotherapy; NED = non-evidence of disease; AWD = alive with disease; DOD = dead of disease; and DOC = dead of other causes.

**Table 3:** Univariate and multivariable analysis of pooled data for OS.

Variable	Univariate Analysis			Multivariable Analysis *		
	HR	95% CI	*p*-Value	HR	95% CI	*p*-Value
Age, years ≤62 >62	0.83 Ref.	0.51–1.36 -	0.47 -			
Sex Male Female	Ref. 1.28	- 0.76–2.16	- 0.35			
cT category 1 2 3 4	Ref. 1.35 2.68 5.76	- 0.59–3.05 1.35–5.25 3.06–10.8	- 0.47 0.004 <0.001	Ref. 1.33 2.65 5.51	- 0.58–3.02 1.34–5.22 2.76–10.5	- 0.5 0.005 <0.001
N category N0 N+	Ref. 1.71	- 1.05–2.80	- 0.03	Ref. 1.09	- 0.66–1.80	- 0.74
Type of surgical resection SR LTBR STBR/TTBR	Ref. 1.78 4.65	- 0.71–4.50 1.86–11.6	- 0.22 0.001			
Surgical margins Negative Positive	Ref. 2.38	- 1.36–4.16	- 0.002			
Adjuvant treatments None RT/CRT	Ref. 1.60	- 0.99–2.55	- 0.052			

* Calculated on 274 cases.HR = hazard ratio; CI = confidence interval; Ref. = reference category; SR = sleeve resection; LTBR = lateral temporal bone resection; STBR = subtotal temporal bone resection; TTBR = total temporal bone resection; RT = radiotherapy; and CRT = chemoradiotherapy.

**Table 4:** Univariate and multivariable analysis of pooled data for DSS.

Variable	Univariate Analysis			Multivariable Analysis *		
	HR	95% CI	*p*-Value	HR	95% CI	*p*-Value
Age, years ≤62 >62	1.20 Ref.	0.71–2.05 -	0.49 -			
Sex Male Female	Ref. 1.37	- 0.78–2.40	- 0.27			
cT category 1 2 3 4	Ref. 8.99 24.4 58.8	- 1.08–74.7 3.29–181.1 8.10–426.4	- 0.042 0.002 <0.001	Ref. 8.90 23.8 56.0	- 1.07–73.9 3.20–177.8 7.67–409.1	- 0.043 0.002 <0.001
N category N0 N+	Ref. 1.97	- 1.13–3.42	- 0.017	Ref. 1.07	- 0.61–1.87	- 0.81
Type of surgical resection SR LTBR STBR/TTBR	Ref. 1.85 6.85	- 0.56–6.10 2.13–22.0	- 0.31 0.001			
Surgical margins Negative Positive	Ref. 3.55	- 1.85–6.84	- <0.001			
Adjuvant treatments None RT/CRT	Ref. 2.33	- 1.26–4.31	- 0.007			

* Calculated on 271 cases.HR = hazard ratio; CI = confidence interval; Ref. = reference category; SR = sleeve resection; LTBR = lateral temporal bone resection; STBR = subtotal temporal bone resection; TTBR = total temporal bone resection; RT = radiotherapy; and CRT = chemoradiotherapy.

## Data Availability

Not applicable.

## Supplementary Materials

The following supporting information can be downloaded at: https://www.mdpi.com/article/10.3390/jcm12072490/s1, Table S1: PRISMA checklist; Table S2: Quality assessment score for each included study, based on the Newcastle-Ottawa scale (NOS); Figure S1: Kaplan–Meier curves of OS and DSS for the pooled sample; Figure S2: Kaplan–Meier curves of OS according to che cT caterogy (a), and the N status (b); Figure S3: Funnel plots for the analyses of cT category and overall survival (a), cT category and disease specific survival (b), N status and overall survival (c), N status and disease specific survival (d).
