# Supplementary material for: Survival Outcomes in Squamous Cell Carcinoma of the External Auditory Canal: A Systematic Review and Meta-Analysis
Source: J Clin Med. 2023 Mar 24;12(7):2490. doi: 10.3390/jcm12072490 (PMC10094887; doi:10.3390/jcm12072490)
Supplement: Supplementary file 1 [file jcm-12-02490-s001.zip › Table S2.pdf]

Table S2. Quality assessment score for each included study, based on the Newcastle-Ottawa scale (NOS).

Author	Selection				Comparability	Outcome			Total
	Representativeness of the exposed cohort	Selection of the non-exposed cohort	Ascertainment of exposure	Demonstration that outcome of interest was not present at start of study	Comparability of cohorts on the basis of the design or analysis	Assessment of outcome	Was follow-up long enough for outcomes to occur	Adequacy of follow up of cohorts	
**Choi et al. [22]**	x	/	x	x	/	x	x	x	6
**Martinez-Devesa et al. [23]**	x	/		x	/			x	3
**Bibas et al. [24]**	x	/		x	/	x	x		4
**Kunst et al. [25]**	x	/	x	x	/	x		x	5
**Chang et al. [26]**	x	/	x	x	/	x	x	x	6
**Ito et al. [27]**	x	/	x	x	/	x	x	x	6
**Bacciu et al. [28]**	x	/	x	x	/	x	x	x	6
**Ouaz et al. [29]**		/	x	x	/	x		x	4
**Zhen et al. [30]**	x	/	x	x	/	x		x	5
**Wierzbicka et al. [31]**	x	/	x	x	/	x	x	x	6
**Park et al. [32]**	x	/	x	x	/	x		x	5
**Ngu et al. [33]**		/	x	x	/	x	x	x	5

**Sajio et al. [34]**	x	/		x	/	x	x	x	5
**Smit et al. [35]**	x	/	x	x	/	x	x	x	6
**Mazzoni et al. [4]**	x	/	x	x	/	x	x	x	6
