# Supplementary figures and images for: Survival Outcomes in Squamous Cell Carcinoma of the External Auditory Canal: A Systematic Review and Meta-Analysis
Source: J Clin Med. 2023 Mar 24;12(7):2490. doi: 10.3390/jcm12072490 (PMC10094887; doi:10.3390/jcm12072490)

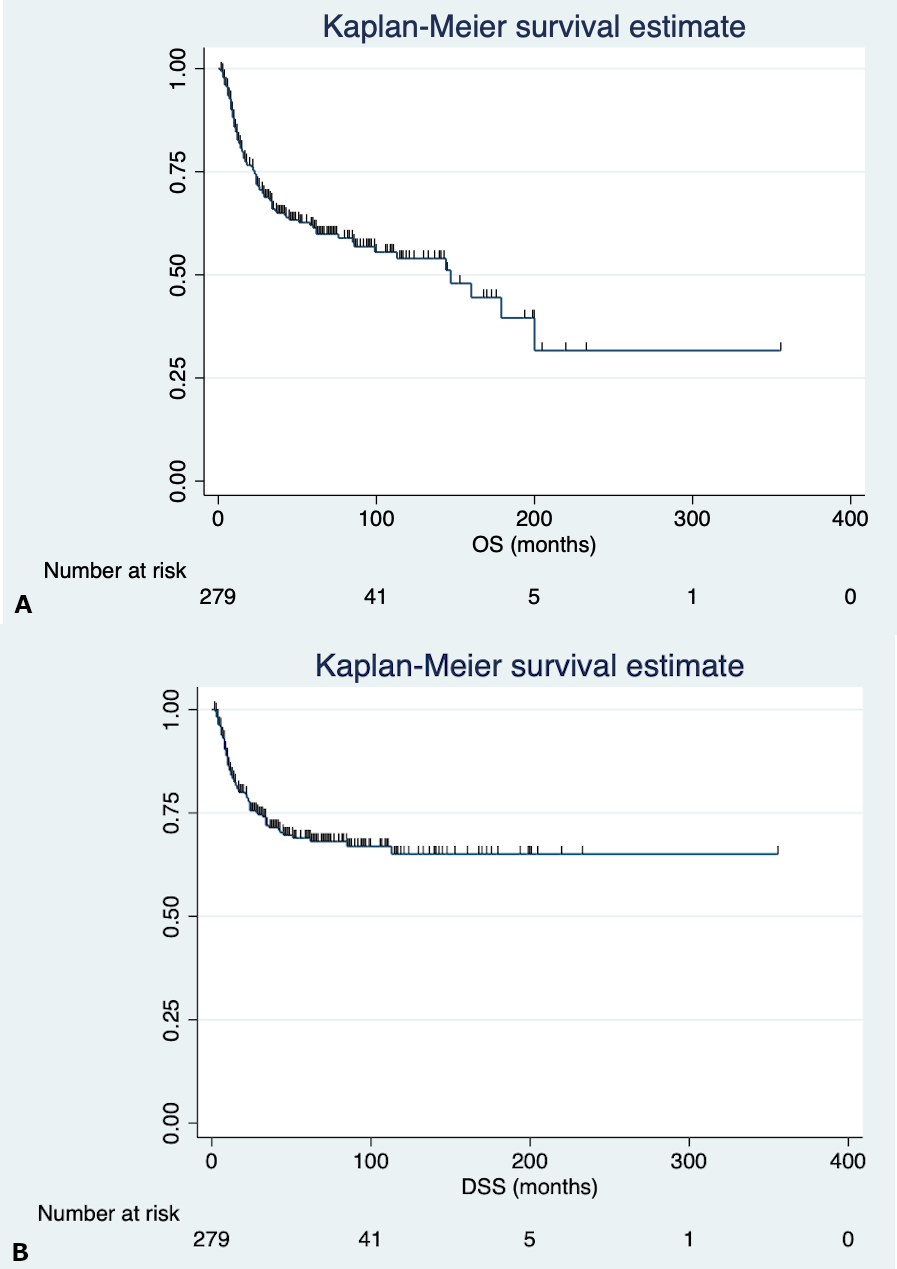

Supplement: Supplementary file 1 [file jcm-12-02490-s001.zip › Figure S1a-b_K-M.tif]

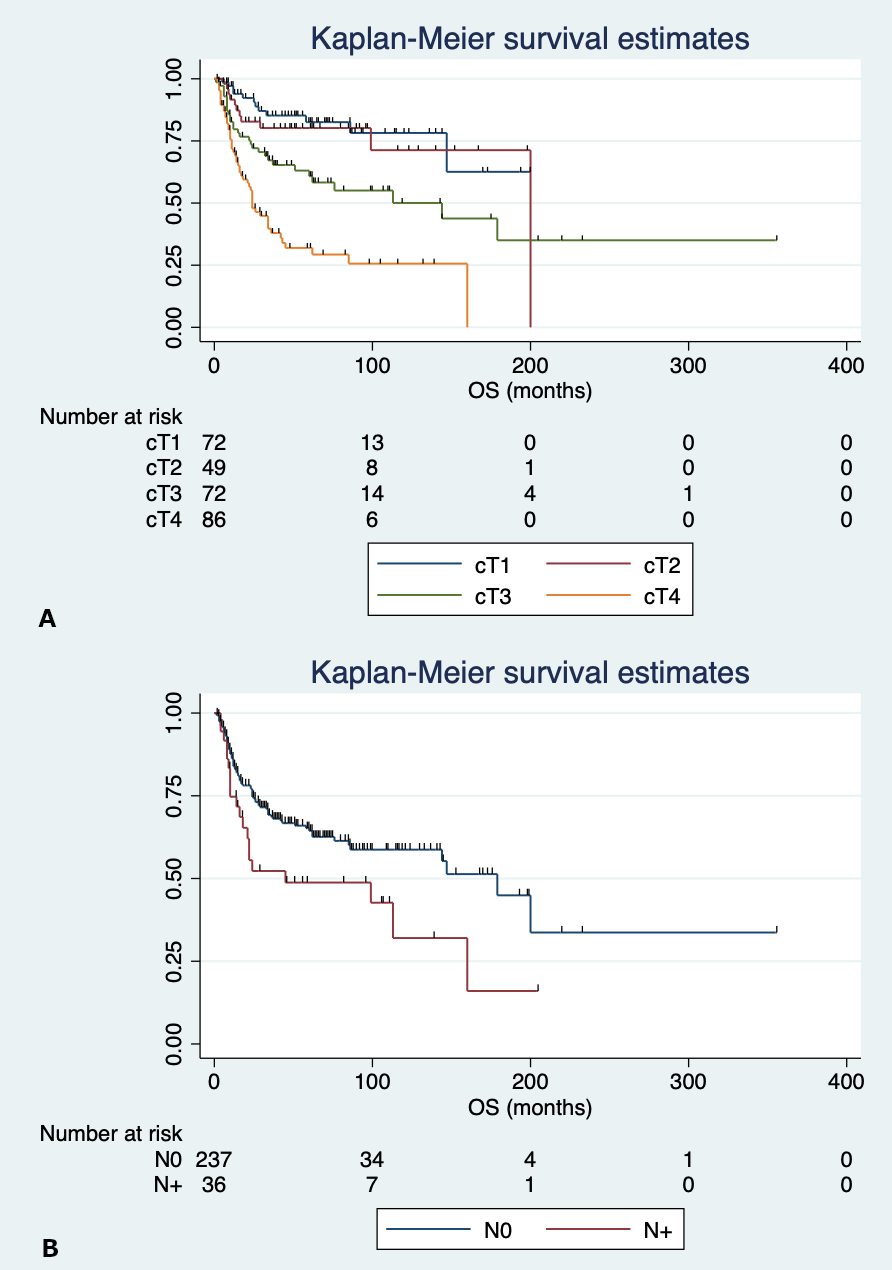

Supplement: Supplementary file 1 [file jcm-12-02490-s001.zip › Figure S2a-b_OS.tif]

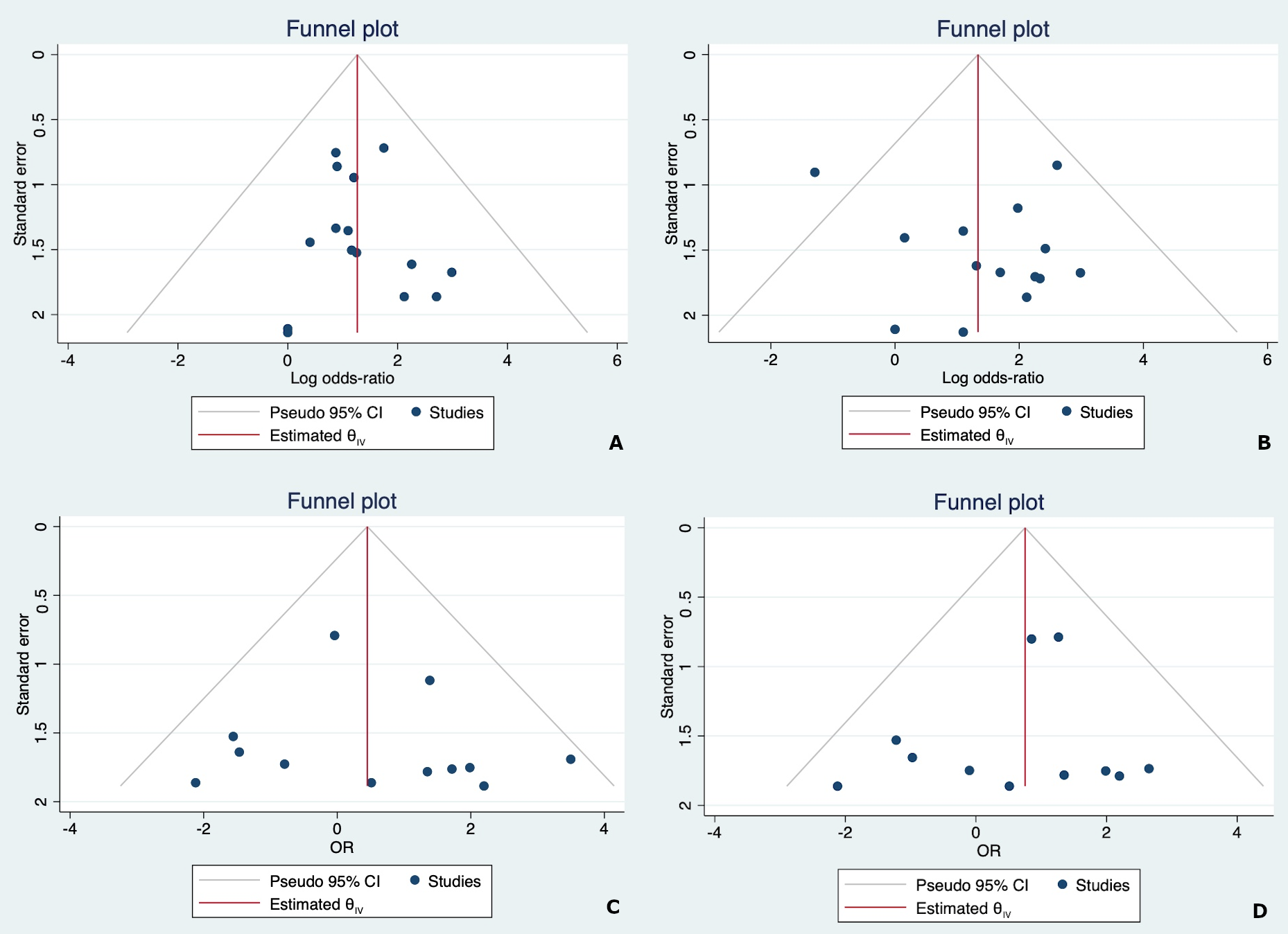

Supplement: Supplementary file 1 [file jcm-12-02490-s001.zip › Figure S3a-b-c-d_Funnel.tif]
